# Firing clamp: a novel method for single-trial estimation of excitatory and inhibitory synaptic neuronal conductances

**DOI:** 10.3389/fncel.2014.00086

**Published:** 2014-03-27

**Authors:** Anton V. Chizhov, Evgenya Malinina, Michael Druzin, Lyle J. Graham, Staffan Johansson

**Affiliations:** ^1^Computational Physics Laboratory, Division of Plasma Physics, Atomic Physics and Astrophysics, A.F. Ioffe Physical-Technical Institute of the Russian Academy of SciencesSt. Petersburg, Russia; ^2^Section for Physiology, Department of Integrative Medical Biology, Umea UniversityUmea, Sweden; ^3^Department of Neurodynamics and Neurobiology, Lobachevsky State University of Nizhny NovgorodNizhny Novgorod, Russia; ^4^Neurophysiology and New Microscopies Laboratory, INSERM U603 - CNRS UMR 8154, Université Paris DescartesParis, France

**Keywords:** synaptic conductance estimation, dynamic clamp, firing-clamp

## Abstract

Understanding non-stationary neuronal activity as seen *in vivo* requires estimation of both excitatory and inhibitory synaptic conductances from a single trial of recording. For this purpose, we propose a new intracellular recording method, called “firing clamp.” Synaptic conductances are estimated from the characteristics of artificially evoked probe spikes, namely the spike amplitude and the mean subthreshold potential, which are sensitive to both excitatory and inhibitory synaptic input signals. The probe spikes, timed at a fixed rate, are evoked in the dynamic-clamp mode by injected meander-like current steps, with the step duration depending on neuronal membrane voltage. We test the method with perforated-patch recordings from isolated cells stimulated by external application or synaptic release of transmitter, and validate the method with simulations of a biophysically-detailed neuron model. The results are compared with the conductance estimates based on conventional current-clamp recordings.

## Introduction

Understanding information processing in the brain requires knowledge of neuronal impulse activity and the corresponding synaptic conductance inputs onto target neurons. In particular, phenomena generated by intracortical interactions, including electrical rhythms, waves, and responses to natural or artificial stimulation, could be better understood if the simultaneous firing activities of excitatory and inhibitory neuron populations were known. Thus, the synaptic conductances arising from these populations, which control the evolution of transmembrane voltage of the target neuron, may provide information on the excitatory and inhibitory neuronal population activities. Estimates of postsynaptic conductances can be obtained from intracellular recordings in a single neuron, but experimental methods of such estimations are still under development, with a principal difference between methods being whether they require repetitive recordings that assume identical input conditions, or not. Given the variability in neuronal responses, even to identical stimuli, single-trial methods are preferable and indeed must be used in some cases, e.g., for evaluation of non-stationary spontaneous activity.

The perhaps most basic method for conductance estimation (Borg-Graham et al., 1998; Anderson et al., 2000; Priebe and Ferster, 2005; Monier et al., 2008; Supplementary Material S1) implies intracellular measurements of membrane voltage or current at different levels of membrane polarization and thus requires repeatable recordings. In *in vivo* conditions, the method is applied to study evoked responses, when the most important information is contained in signals averaged over several trials. Assuming only two types of synaptic input, thus excitatory and inhibitory, if the averaged traces of voltage or current are recorded at different levels of polarization, and the reversal potentials of both excitatory and inhibitory currents (*V*_*E*_ and *V*_*I*_) are known, then algebraic calculations provide estimates for the corresponding conductances, *G*_*E*_ and *G*_*I*_. In the ideal case, it is sufficient to have current records obtained in the voltage-clamp mode at two holding potentials. The difference between the current traces is proportional to the conductance of the target neuron with the difference between the holding potentials as a coefficient. In realistic conditions, the response variability from trial to trial, the contribution of capacitive and voltage-gated currents to the recorded signals, etc. reduce the precision of estimation. In a recent paper, Odom and Borisyuk (2012) generalized the current-clamp approach to the case of three estimated synaptic conductances with the help of multiplicative noise, but with the assumption that non-linear channels do not contribute to the recorded voltage traces and the variance of estimated conductance is known *a priori*.

In the case of non-stationary or on-going activity, a single-trial estimation method is required. The most basic method in this case is by periodically perturbing the membrane potential under current clamp with a train of hyperpolarizing current pulses (Douglas et al., 1988). Samples of the cell input conductance are then derived from the corresponding voltage deflections according to a linear model of the cell. This simple approach has two major limitations. First, the repetition rate of the probe current pulses is limited by the resting time constant of the neuron, which typically has an upper bound of tens of milliseconds, corresponding to a maximum sample rate of tens of hertz. For relatively slow synaptic dynamics, for example those underlying up-and-down states (Leger et al., 2005), this rate may be sufficient, but may not be sufficient to measure rapid transient synaptic inputs, for example as seen during visually-evoked activity. In addition, as with the previous current-clamp based methods, a second limitation is that the estimate does not account directly for the non-linear properties of the membrane, in particular when the cell is firing.

Alternatively, a more sophisticated current-clamp method has been proposed by Rudolph et al. (2004), based on the sensitivity of voltage fluctuations, or “noise,” to the input conductance. In this method the statistical treatment of the recorded membrane voltage is performed with the help of the stationary solution of the Fokker-Planck equation. According to some basic assumptions, this method requires statistically stable states on a time scale of about 100 ms. Thus, for example, the method provides estimates of the up- and down-states of cortical activity *in vivo*, but on the other hand, as above, it is not appropriate for the analysis of faster transient or rhythmic (e.g., theta or gamma range) activity. Similar approaches have been proposed in recent studies (Chizhov and Graham, 2004; Kobayashi et al., 2011). Nevertheless, the question of contamination from spikes and other non-linear responses must still be considered.

The basic principles and limitations of conductance estimation are determined by the control properties of the neuronal membrane. Any synaptically activated ion channel affects the electric activity of the membrane mainly via two mechanisms, shunting and change of polarization due to current. Different synaptic channel types contribute to each of the effects to a different extent, according to their reversal potential and conductance. Their combined effects will determine the total synaptic conductance and total synaptic current. These two signals constitute the principal input signals that control a neuron. This fact is evident from consideration of an equation for membrane voltage according to Kirchhoff's current law (see details in Supplementary Material S2; Pokrovskii, 1978). If a neuron has an arbitrary set of synapses with voltage-independent conductances and reversal potentials, then the voltage equation contains only two types of terms related to input via synapses or electrode, i.e., terms that are linearly dependent on voltage and terms that are voltage-independent [see Equations (A6, A7) in Supplementary Material S2]. The terms of each type determine two linear combinations of input parameters, which control the voltage dynamics. The coefficient of the linearly voltage-dependent term is the total synaptic conductance whereas the voltage-independent term can be referred to as the synaptic current measured at a certain fixed voltage. These two control signals are scalar for one-compartmental and vectorial for multi-compartmental neurons. Because the main assumption requires the synaptic conductances to be voltage independent, such analysis gives only an approximate estimate in the presence of the NMDA-receptor type of glutamatergic channels, which are subject to voltage-dependent block by external Mg^2+^. Nevertheless, with the above stipulation, the control property described above explains that only two linear combinations of input variables such as the total synaptic conductance and total synaptic current are required to control the voltage. An important consequence following from the given assumptions is that only two input conductances may be estimated using the characteristics of the voltage trace (see also in Odom and Borisyuk, 2012). However, it should be noted that extra assumptions on the temporal characteristics of the synaptic conductances may allow further splitting of the input signals as, for example, in the multi-trial variant of (Odom and Borisyuk, 2012) with extra limitations assumed for the conductance fluctuations.

Simultaneous estimation of two input signals requires conditions in which the voltage evolution is sensitive to changes of current as well as conductance. Such sensitivity is present during spiking, because the mean subthreshold potential is primarily sensitive to the magnitude of current whereas the spike amplitude is more affected by the shunting effect of the total conductance on the depolarization induced by the sodium current, as will be seen from our recordings and simulations. This gives rise to the idea that *G*_*E*_ and *G*_*I*_ may be estimated if the spiking regime could be maintained. The temporal resolution of such estimation will be determined by the frequency of spikes within the train. The precision will be dependent on the possibilities to minimize the contaminating effects of intrinsic ionic channels, noise, experimental artifacts, and synaptic conductance changes between the spikes. Importantly, the most significant of them, the effect of intrinsic ionic channels, might be eliminated if one provides constant interspike interval and after-spike voltage reset, e.g., impose strict initial conditions on the cell prior to each imposed action potential.

To improve the single-trial estimation of synaptic activity, we propose a new quasi-dynamic clamp method that specifically exploits the non-linear dynamics of the action potential. Thus, *in lieu* of a train of stereotyped hyperpolarizing current pulses, as in the previous single-trial method, here the sample probes are spikes evoked by a train of bi-phasic meander-like current stimuli. The probe spikes are evoked at a constant frequency, and therefore we call the method “firing clamp.” Here we describe the method, and present results from electrophysiological recordings of isolated neurons *in vitro*, where conductance responses are evoked by external application or synaptic release of neurotransmitter. In the Supplementary Material we also present results from numerical simulations of a neuron model for validating the technique.

## Methods

### Dissociated cell preparation and electrophysiology

Preparation of thin (300 μm) coronal brain slices obtained from young (4–5 weeks) male Sprague–Dawley rats and mechanical dissociation of medial preoptic neurons from the anterior hypothalamic area as well as composition of extra- and intracellular experimental solutions are described in (Druzin et al., 2011). The extracellular solution, without or with test reagents, was applied by a gravity-fed fast perfusion system. All experiments were carried out at room temperature, 21–23°C. Whole-cell amphotericin-B perforated-patch recordings were made using a Multiclamp 700A amplifier (Molecular Devices, USA) and an acquisition card NI-PCI-6221 (National Instruments, USA) which together with a dual-core Intel processor under Windows XP provided a dynamic clamp time step of 30 μs, using custom software (DC_Project.exe at http://www.ioffe.ru/CompPhysLab/AntonV3.html). The injected current was set as *I*_*inj*_(*t*) = *I*(*t*) − *G*(*t*) (*V*(*t*) − *V*_0_), where *I*(*t*) and *G*(*t*) are the simulated input current and conductance, respectively, and *V*_0_ is the resting membrane potential. In the firing-clamp regime the current included the meander current, described further below.

### “Firing-clamp”

#### Background protocol

**Figure 1:**
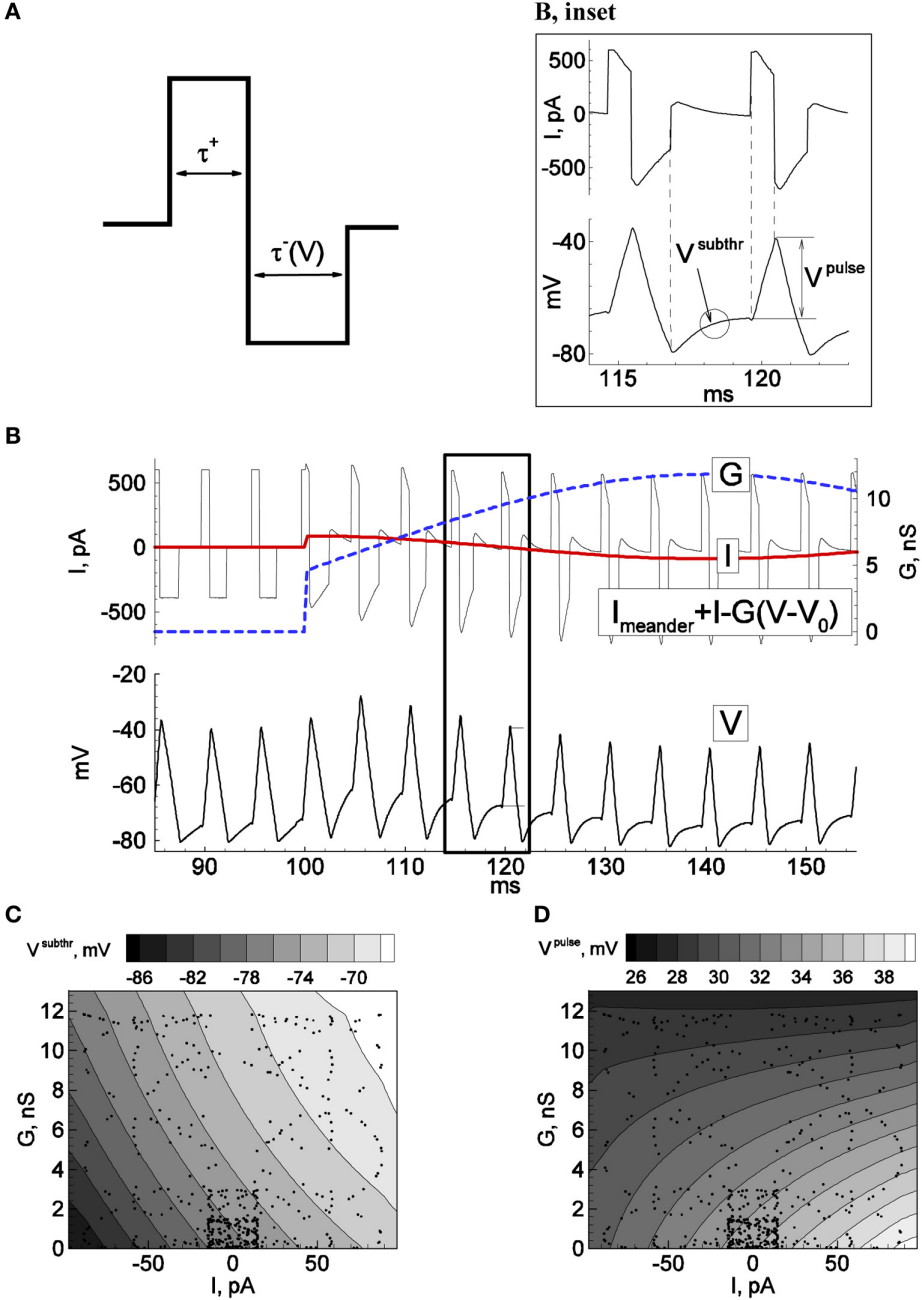
**Principle of firing-clamp technique for conductance estimation: Calibration procedure**. Recordings from an MPN neuron were performed in perforated-patch configuration. By means of real-time computer control (“firing-clamp”) the voltage-dependent meander-wise current shown in **(A)** was repeatedly injected at a maintained rate, 200 Hz, which evoked probe spikes (**B**, bottom). The positive and negative pulse amplitudes of the meander were 0.4 and –0.3 nA, respectively. The positive pulse duration τ^+^ was fixed at 0.81 ms, and the negative pulse duration τ^−^ (*V*) was controlled by the recorded membrane voltage, with pulse termination when the voltage reached –80 mV. During calibration, additional sinusoidal current *I* and conductance *G* were generated with the dynamic-clamp system, with periods of 40 and 70 ms, respectively. The subthreshold voltage *V*^*subthr*^ and the positive pulse response amplitude *V*^*pulse*^ were measured for each probe cycle and plotted as functions of the injected *I* and *G*. The spikes correspond to dots in **(C)** and **(D)**. The functions were approximated by the quadratic polynomials *V*^*subthr*^(*I*, *G*) = 0.002 *I G* + 0.079 *I* + 0.8 *G* − 76.8, and *V*^*pulse*^(*I*, *G*) = −0.0039 *I G* + 0.0477 *I* − 0.49 *G* − 34.9, where voltage, current, and conductance are given in mV, pA, and nS, respectively.

The regime of constant-rate probe-spike firing is set by the injection of a meander-shaped current shown in Figures 1A,B, with a rate of 200 Hz. The positive and negative pulse amplitudes depend on the cell admittance and, for the example shown in Figure 1, were chosen to be 600 and −400 pA for the neuron with input conductance of approximately 1.5 nS. The positive pulse duration was fixed at 0.81 ms. The negative pulse was maintained until the recorded voltage crossed the fixed reset value –75 mV. We denote the meander current as *I*_*Meander*_ (Figure 1B).

#### Calibration

The calibration procedure is aimed to obtain the functions *V*^*subthr*^(*I*, *G*) and *V*^*pulse*^(*I*, *G*) shown in Figures 1C,D. Practically, it is enough to get a few tens of *V*^*subthr*^ and *V*^*pulse*^ values corresponding to sparsely distributed points (see dots) in a physiologically meaningful domain of the *I*-*G*-plane. The functions *V*^*subthr*^(*I*, *G*) and *V*^*pulse*^(*I*, *G*) are then obtained by the least-square method as a quadratic polynomial approximation.

#### Recordings

The target recordings are carried out in the same conditions as during the calibration, i.e., in the presence of meander-like current injection.

#### Data analysis

At each probe spike *i* of the recorded voltage, *V*^*subthr*^_*i*_ and *V*^*pulse*^_*i*_ are measured. The system of equations *V*^*subthr*^_*i*_ = *V*^*subthr*^(*I*_*i*_, *G*_*i*_) and *V*^*pulse*^_*i*_ = *V*^*pulse*^(*I*_*i*_, *G*_*i*_) was then solved using the approximations obtained from the calibration. Considering only excitatory and inhibitory synapses with reversal potentials *V*_*E*_ and *V*_*I*_, the input signals (*I*_*i*_, *G*_*i*_) are transformed into the synaptic conductances for each probe spike (*G*_*E*,*i*_, *G*_*I*,*i*_) as follows (see also Supplementary Material S2):

$$G_{E,i}=(I_{i}-G_{i}(V_{I}-V_{0}))/(V_{E}-V_{I}),\;G_{I,i}=G_{i}-G_{E,i}.$$

## Results

As stated in the Introduction, estimation of excitatory and inhibitory synaptic conductances, or equivalently, the input current and conductance, can be performed using probe spikes (Figure 1B). To obtain a fixed spike frequency we injected current of a meander-like shape (Figure 1A) repeated at a fixed rate, according to the protocol “firing-clamp” described in Methods. Under firing clamp, the initial phase of each meander pattern is comprised of a depolarizing (positive) current pulse of fixed duration (Figure 1A), which leads to the suprathreshold activation of sodium channels and a spike. The positive current pulse is then followed immediately by a hyperpolarizing (negative) current pulse, which lasts until the voltage crosses a defined reset value (normally set at –75 mV) (the fact that the duration of this pulse is a function of the measured voltage distinguishes the recording from a strict current-clamp configuration, and thus being formally a dynamic-clamp recording mode). For the remainder of the measurement cycle the injected current is set to zero.

The response to this stimulus pattern provides several conditions for probing the conductance state of the neuron, according to the three key ideas of the firing-clamp approach. First, the fast dynamics of the action potential, much faster than the “resting” membrane time constant, allow a rapid sampling rate, e.g., 100–200 Hz. Second, the imposed reset by the hyperpolarizing current pulse ensures that the states of the neuron's fast voltage-dependent sodium and potassium channels are approximately identical at each cycle, as well as allowing a fast spike rate due to an imposed de-inactivation of the sodium channels. Finally, the fixed firing frequency ensures a near constant state for the slow calcium- and voltage-dependent channels (typically potassium) that underlie spike frequency adaptation under normal firing.

This non-linear method suggests an emphasis on measures that are distinctly sensitive to synaptic current and to synaptic conductance. Thus, for our purposes it is convenient to express the total synaptic input in terms of a pure shunting component, *G*(*t*), and a pure current component, *I*(*t*), the latter measured from the resting potential *V*_0_ (see details in Supplementary Material S2; Pokrovskii, 1978). For the case of only two, excitatory and inhibitory synaptic types, the input signals are:

$$\begin{array}{l} G(t)=G_{E}(t)+G_{I}(t)\\ I(t)=G_{E}(t)(V_{E}-V_{0})+G_{I}(t)(V_{I}-V_{0}) \end{array}$$

Note that the *G*(*t*) contributes a linear term of the membrane voltage *V*(*t*) in the membrane current equation, and that *I*(*t*) contributes a term independent of *V*(*t*) [see Equations (A6, A7) in Supplementary Material S2].

The probing by spikes is well-suited to estimate the two synaptic components (Figure 1B, inset). In particular, the positive pulse response voltage *V*^*pulse*^, defined as the voltage difference at the beginning and end of the positive current pulse, is mainly affected by the shunting effect of the total synaptic conductance. Conversely, the mean subthreshold potential *V*^*subthr*^ between spikes after the imposed reset, thus during the inter-meander interval when no current is injected, is primarily sensitive to the magnitude of synaptic current (Figures 1C,D). However, these relations are not known a priori for a given neuron. Therefore, before estimating conductance changes from intrinsic synapses, a dynamic-clamp calibration that injects artificial synaptic conductance waveforms to the neuron during quiescent conditions is necessary to estimate the relationships between these voltage measures and the synaptic state:

$$\begin{array}{l} V^{pulse}=V^{pulse}(I,G)\\ V^{subthr}=V^{subthr}(I,G) \end{array}$$

The precision of the estimation depends on the possibility to control the impact of intrinsic ionic channels, with the implicit assumption that the bandwidth of the synaptic conductance changes is consistent with the sampling rate of the probe spikes, as well as the standard concerns of noise and experimental artifacts (e.g., due to imperfect electrode compensation). Importantly, the most significant factor, the effect of intrinsic ionic channels, is constrained by enforcing a constant interspike interval and after-spike voltage reset, imposing strict initial conditions on the cell during each measurement cycle.

### “Firing clamp” in simulations

To validate the principle underlying the method, we first studied the effects of the stimulation parameters and of noise on the estimation of synaptic input using simulations of a biophysical neuron model, described in the Supplementary Material (Supplementary Material S2). In the case of synaptic conductance oscillations at gamma- or theta-range frequencies, the simulations showed that the firing-clamp separates the excitatory and inhibitory components well and reveal the oscillations. The excitatory conductance estimations are quite precise for both gamma and theta oscillations. The inhibitory conductance estimates tend to be dispersed around the true solution in the shorter time scale case. The method is robust to noise.

### “Firing clamp” in experiments *in vitro*

**Figure 2:**
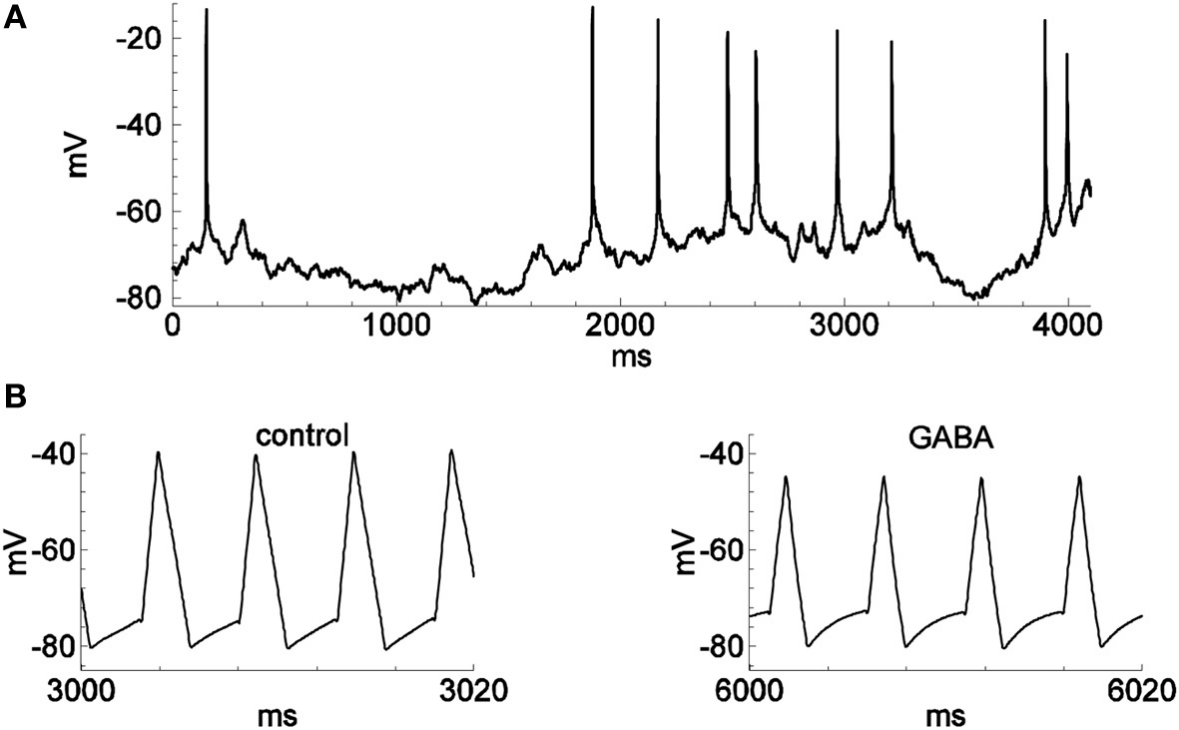
**Spontaneous spiking and the activity in firing-clamp regime during GABA application**. Recordings were from the same neuron as in Figure 1. **(A)** Membrane voltage with occasional spontaneous spikes during hyperpolarizing stimulation by a constant injected current of −30 pA. **(B)** Voltage trace (note expanded time scale) with probe spikes before (left) and during GABA application (1.0 mM; right).

We tested the firing-clamp method with electrophysiological perforated-patch recordings from dissociated neurons *in vitro*. Voltage responses of a sample neuron to steady and meander-like current injection, with and without the application of GABA, are shown in Figures 1, 2 (Karlsson et al., 2011). To obtain a fixed spike frequency of the probe spikes (Figure 1A), we injected meander-like current (Figure 1B) at a fixed rate of 200 Hz (see Methods). We then measured *V*^*subthr*^ and *V*^*pulse*^ for each probe spike (Figure 1B, inset).

To solve the reverse problem of estimating the synaptic input from the measured values of *V*^*subthr*^ and *V*^*pulse*^, the dependence of these variables on *I* and *G* was determined by the calibration procedure (Figures 1C,D) described above, during which a slow oscillating “synaptic” input conductance was generated in the dynamic-clamp recording configuration. The dynamic-clamp current *I*_*inj*_(*t*) was calculated as:

$$I_{inj}(t)=I(t)-G(t)(V(t)-V_{0})$$

This current was then added to the meander-like stimulus current. Practically, we found that it is sufficient to obtain a few hundred values of *V*^*subthr*^ and *V*^*pulse*^ corresponding to sparsely distributed points in a physiologically meaningful domain of the *I*-*G*-plane (see dots in Figures 1C,D). The global dependencies of *V*^*subthr*^ and *V*^*pulse*^ on *I* and *G* were then approximated by the least-square method as quadratic polynomials.

After recording responses to unknown stimuli, the reverse problem to find *I*_*i*_ and *G*_*i*_ for each pair of measured *V*_*subthr*,*i*_ and *V*_*pulse*,*i*_ values at each probe spike *i* was accomplished using the approximations obtained by the calibration. Note that the estimations of *I*_*i*_ and *G*_*i*_ are performed without any assumption on the number of synaptic types nor on their reversal potentials. However, the mapping of each estimated pair of *I*_*i*_, *G*_*i*_ values to the excitatory and inhibitory conductances, *G*_*iE*_, *G*_*iI*_ assumes only two types of receptors with known reversal potentials *V*^*E*^, *V*^*I*^. Specifically, we assume that the responses correspond to AMPA and GABA_*A*_ receptors, respectively. The values of *G*_*E*,*i*_ and *G*_*I*,*i*_ are obtained as:

(1) $$\begin{array}{l} G_{E,i}=\frac{I_{i}-G_{i}(V_{I}-V_{0})}{V_{E}-V_{I}},\\ G_{I,i}\;=G_{i}-G_{E,i}. \end{array}$$

**Figure 3:**
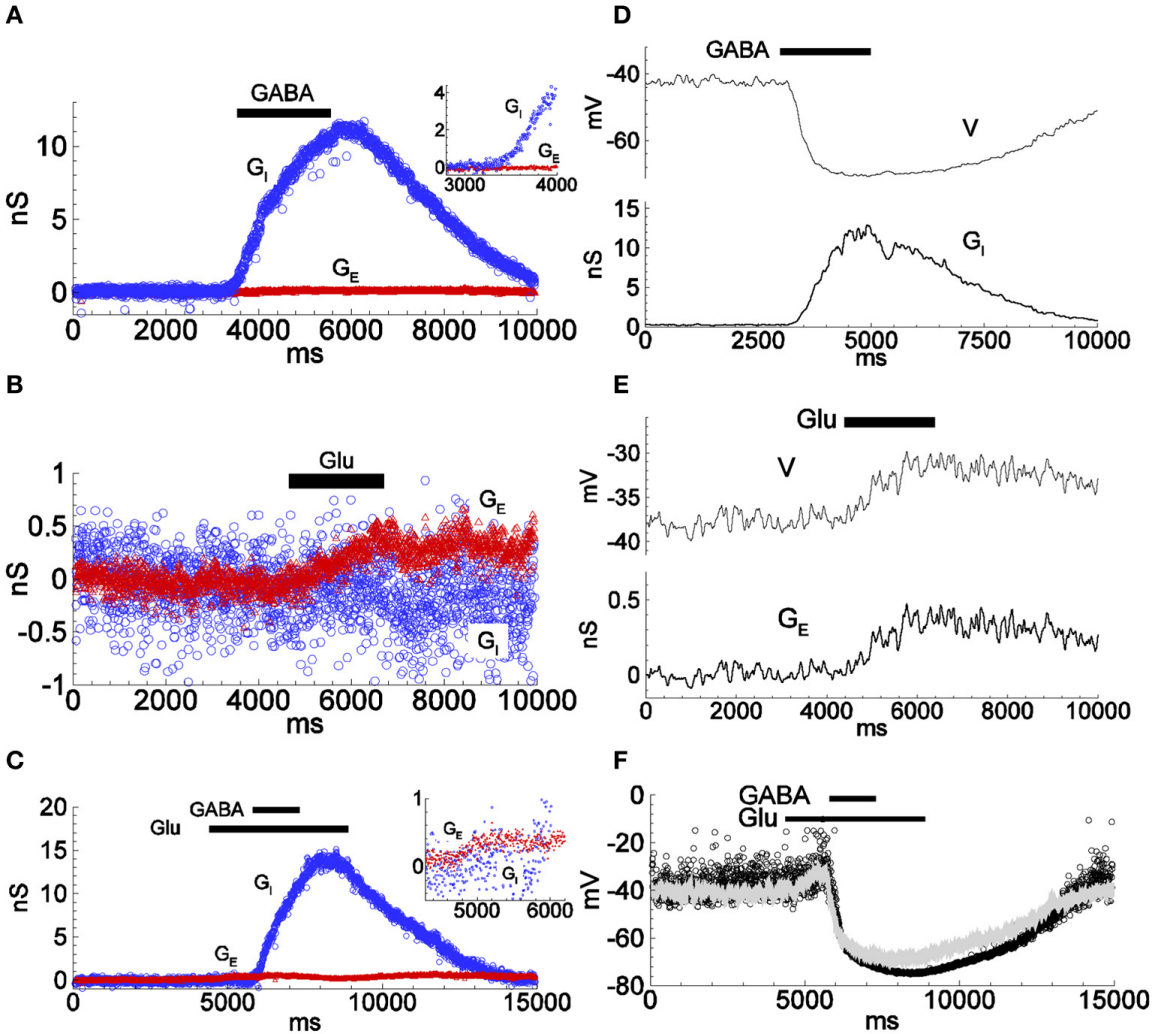
**Conductance estimations for responses to applied inhibitory and excitatory agonists using the firing-clamp and the continuous current-clamp methods. (A–C)** Inhibitory and excitatory conductances were estimated by the firing-clamp method from recordings with extracellular application of either GABA (1.0 mM) (**A**; see also Figure 2), glutamate (Glu; 1.0 mM) **(B)**, or overlapping application of both agonists (**C**; inset highlights the excitatory component in response to glutamate) Each pair of subthreshold voltage and spike amplitude values evaluated at each probe spike was converted into a pair of excitatory and inhibitory conductances (*G*_*E*_, *G*_*I*_), according to the functions shown in Figures 1C,D, given reversal potentials *V*_*E*_ = 0 and *V*_*I*_ = −74 mV. **(D,E)** Conductance estimates (bottom traces in **D**,**E**) from repeated continuous current-clamp recordings of membrane voltage at different holding currents. Inhibitory (**D**, top) and excitatory (**E**, top) voltage responses to application of either GABA (1.0 mM) **(D)**, or glutamate (1.0 mM) **(E)** (see Supplementary Material, for the continuous current-clamp technique of estimation, Equations S1.A1, S1.A2). **(F)** Reconstructed change in membrane voltage from the conductances estimated by the continuous current-clamp method during complex stimulation with glutamate and GABA as in **(C)**, thus as would be expected in the absence of firing clamp (dots), calculated as *V* = (*G*_0_*V*_0_ + *G*_*E*_*V*_*E*_ + *G*_*I*_*V*_*I*_)/(*G*_0_ + *G*_*E*_ + *G*_*I*_). Note the similarity to the voltage response to the same type of stimulation as recorded in conventional current-clamp mode with no injected current, (superimposed gray line). All data from the same cell. The input conductance was 1.5 nS. The traces in **(D,E)** are low-pass filtered. See Supplementary Figure 7, for an example of a cell with larger conductance response to applied glutamate.

We then recorded responses of the dissociated neurons to the application of GABA and/or glutamate (Glu) (Figures 2B, 3). As expected, the estimated GABA-evoked conductance response (Figure 3A) consists of a large *G*_*I*_ component without a *G*_*E*_ component (inset), whereas for glutamate-evoked responses (Figure 3B), the *G*_*E*_ component dominates and the *G*_*I*_ component is negligible (see also Supplementary Figure 7, for the analysis of the glutamate response in another cell). Stimulation by a complex sequence of agonist application (Glu, then Glu + GABA, then Glu, as indicated by bars in Figure 3C) reveals a prominent excitatory conductance component followed by an inhibitory conductance component, with reduced conductance upon washout of agonists, as expected. During long-lasting application of GABA the reversal potential *V*_*I*_ changes (Karlsson et al., 2011). We took into account such changes by introducing a variable *V*_*I*_ in Equation (1), according to the Supplementary Material S4.

We then estimated the evoked conductances under the same conditions with the continuous current-clamp method on repeated trials at different levels of polarization, described previously. The time course and magnitude of the conductances *G*_*E*_ and *G*_*I*_ estimated by the firing-clamp method compare well to those estimated from the continuous current-clamp method (Figures 3D,E). Moreover, the membrane voltage reconstructed from the estimated conductances (Figure 3F, dots) is similar to the voltage response recorded in current-clamp mode with no injected current (Figure 3F, gray line). Taken together, these results attest to the consistency of the different methods for the estimation of the synaptic input during application of inhibitory and/or excitatory agonists.

**Figure 4:**
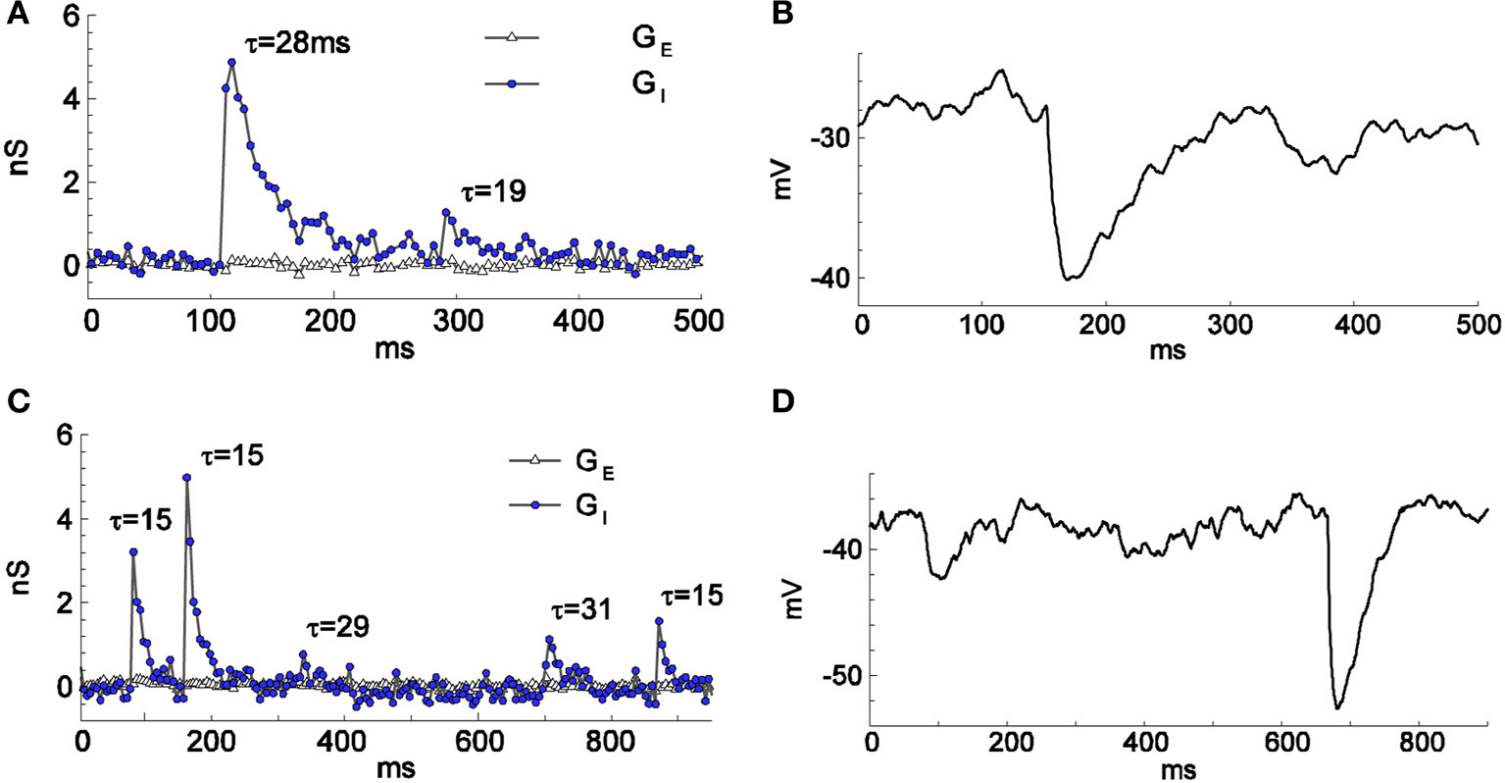
**Spontaneous GABA-mediated postsynaptic events of different amplitudes and time courses recorded in firing-clamp (A,C) and current-clamp (B,D) regimes**. Recordings from two cells (one in **A,B** and another in **C,D**) different from the cell of Figures 1, 2. The resting input conductance was 1.6 nS for the cell in **(A,B)** and 1.1 nS for the cell in **(C,D)**. The time constants (in ms) of mono-exponential fit are given for each event in **(A)** and **(C)**.

To verify that the firing-clamp method is applicable to rapidly changing synaptic conductances, we then estimated the changes in the synaptic conductances during spontaneous, presumably GABA-mediated, synaptic events (Figure 4; recordings from different cells than in Figures 1–3). We recorded a few synaptic events in firing-clamp mode (Figures 4A,C) and, shortly thereafter, in current-clamp mode (Figures 4B,D). The shapes of the estimated inhibitory conductance events are consistent with the shapes of the postsynaptic potentials, demonstrating that the time resolution of the firing-clamp approach is sufficient to reveal isolated synaptic events. In addition, using artificial oscillatory *G*_*E*_ and *G*_*I*_ inputs mimicked by the dynamic clamp, simulations (Supplementary Material S2) and in *in vitro* experiments in brain slices (Supplementary Material S3) demonstrated that the firing-clamp method can reconstruct input conductances changing in the gamma-range frequency.

## Discussion

We have developed a new method, the firing-clamp method, for estimating two types of synaptic input to a recorded neuron, in the experiments described here, comprised of glutamatergic input, presumably mediated by AMPA receptors, and GABAergic input, presumably mediated by GABA_A_ receptors. The method is validated with simulations of a biophysical neuron model, demonstrating that the method performs well with transient and noisy synaptic inputs (see Supplementary Material S2). Experiments using *in vitro* recordings of isolated neurons and in the brain slice show that the method allows the extraction of inhibitory only, excitatory only, and combined responses to applied synaptic agonists, as well as conductance changes underlying fast spontaneous activity. The (preoptic) neurons studied here are known to express voltage-gated Na^+^, K^+^, and Ca^2+^ channels and GABA_A_-, AMPA-, and NMDA-receptors of types similar to those found in a majority of central neurons. Their detailed biophysical properties, however, vary between cells (c.f. legends to Figures 3, 4 and Supplementary Figure 7 for the input conductance difference) and also from the model, which was based on hippocampal neurons, demonstrating that the firing-clamp method is applicable for any neuronal type. Comparing with the one-trial method from (Rudolph et al., 2004), the bandwidth of the estimated conductances from the proposed firing-clamp is improved by a factor of 20 or more. An alternative single-trial estimation approach from Paninski et al. (2012) provides a temporal resolution of tens of milliseconds, but has not yet been applied for resolving both excitatory and inhibitory components in experiments.

The sensitivity of the method is due to the fact that the crucial measured characteristics of the probe spikes, *V*^*subthr*^ and *V*^*pulse*^, depend on the total synaptic input current measured from rest, *I*, and the total synaptic conductance, *G*, in different ways. As shown in Figures 1C,D, over much of the relevant range of inputs, *V*^*subthr*^ depends mostly on *I* whereas *V*^*pulse*^ is more sensitive to *G*.

The underlying assumptions limit the applicability of the firing clamp method in its present version to cases when synaptic conductances can be considered as voltage independent. However, further development of the method may be envisaged, for example, introducing a third characteristic of the probe spikes, sensitive to the known voltage dependence of NMDA-receptor type glutamatergic excitatory synapses.

In conclusion, the proposed method can be used to estimate non-stationary synaptic activity, including single synaptic events, underlying neuronal population interactions and is likely to be useful in *in vivo* conditions for studies of oscillating activity in the gamma or theta range, epileptic discharges, etc.

## Author contributions

Anton V. Chizhov worked on method formulation, programming, simulations, experiments and paper writing. Michael Druzin and Staffan Johansson did experiments and paper writing. Evgenya Malinina did experiments. Lyle J. Graham stated the problem and worked on paper writing.

### Conflict of interest statement

The authors declare that the research was conducted in the absence of any commercial or financial relationships that could be construed as a potential conflict of interest.
