# Corrigendum: Firing clamp: a novel method for single-trial estimation of excitatory and inhibitory synaptic neuronal conductances

**DOI:** 10.3389/fncel.2014.00149

**Published:** 2014-05-28

**Authors:** Anton V. Chizhov, Evgenya Malinina, Michael Druzin, Lyle J. Graham, Staffan Johansson

**Affiliations:** ^1^Computational Physics Laboratory, Division of Plasma Physics, Atomic Physics and Astrophysics, A.F. Ioffe Physical-Technical Institute of the Russian Academy of SciencesSt. Petersburg, Russia; ^2^Department of Integrative Medical Biology, Umea UniversityUmea, Sweden; ^3^Department of Neurodynamics and Neurobiology, Lobachevsky State University of Nizhny NovgorodNizhny Novgorod, Russia; ^4^Neurophysiology and New Microscopies Laboratory, Centre National de la Recherche Scientifique, Université Paris DescartesParis, France

**Keywords:** synaptic conductance estimation, dynamic clamp, firing-clamp, neuron models, neurophysiology

It is important to note that Bédard and colleagues have also described a method for the single trial extraction of synaptic input (Bédard et al., 2012). This method, which requires that the membrane voltage remains subthreshold, thus in a linear domain, obtains an estimate for synaptic inputs based on time derivatives of passive recordings of the membrane voltage trace, i.e., without perturbations from injected current by the amplifier.

## Conflict of interest statement

The authors declare that the research was conducted in the absence of any commercial or financial relationships that could be construed as a potential conflict of interest.
