# Supplementary material for: Firing clamp: a novel method for single-trial estimation of excitatory and inhibitory synaptic neuronal conductances
Source: Front Cell Neurosci. 2014 Mar 27;8:86. doi: 10.3389/fncel.2014.00086 (PMC3973923; doi:10.3389/fncel.2014.00086)
Supplement: Supplementary file 1 [file Presentation1.PDF]

Supplementary material

S1. Multi-trial continuous conductance estimation method in current-clamp mode

The conventional multi-trial method of continuous conductance estimation was originally proposed using the voltage-clamp recording mode by Borg-Graham et al. (1998) and subsequently in modified form using the current-clamp recording mode by Priebe and Ferster (2005). In the current-clamp version, the method uses stimulus-evoked voltage responses that are recorded at two or more levels of injected constant current. It is assumed that the voltage dynamics can be described by a deterministic point-neuron model in the absence of active conductances, allowing the estimation excitatory and inhibitory conductances at each moment in time (thus, determined by the sampling rate of the data acquisition). The voltage-clamp and current-clamp variations have been used in a variety of *in vivo* and *in vitro* studies, for example in the cat visual cortex (Borg-Graham et al., 1998; Priebe and Ferster 2005; Anderson et al., 2000). For the current-clamp approach, the equations written for two measured voltage traces, $V^1(t)$ and $V^2(t)$, corresponding to two levels of holding current (taken here as equal to zero and a non-zero value I), are as follows:

$$-G_E(t)(V^1(t)-V_E)-G_I(t)(V^1(t)-V_I)-G_0(V^1(t)-V_0)=0$$

$$-G_E(t)(V^2(t)-V_E)-G_I(t)(V^2(t)-V_I)-G_0(V^2(t)-V_0)+I=0,$$

where I is the constant current applied when measuring $V^2(t)$. The system of equations gives rise to the estimations:

$$G_I=\frac{-G_0\left[(V^1-V_0)(V^2-V_E)-(V^2-V_0)(V^2-V_E)\right]-I(V^1-V_E)}{(V^1-V_I)(V^2-V_E)-(V^2-V_I)(V^2-V_E)}, \tag{A1}$$

$$G_E=\frac{-G_0\left[(V^1-V_0)(V^2-V_I)-(V^2-V_0)(V^2-V_I)\right]-I(V^1-V_I)}{(V^1-V_E)(V^2-V_I)-(V^2-V_E)(V^2-V_I)} \tag{A2}$$

S2. Conductance estimation method "firing clamp" in simulations

Mathematical model of a neuron and its input signals

We consider a conductance-based Hodgkin-Huxley type model of an adaptive hippocampal pyramidal neuron with ionic currents I_{Na}, I_{DR} , I_A , I_H , I_M (Borg-Graham, 1998) and I_{AHP} (Kopell et al., 2000). The calcium-dependent potassium current I_{AHP} provides slow adaptation and the potassium current I_M provides fast adaptation. The explicit formulation of the model can be found in (Chizhov and Graham, 2007). The membrane area is set to $2{\cdot}10^{-5}$ cm^2. The external input may consist of different types of synaptic currents and the current through the electrode I_a, i.e.

$$I_{ext}(t,V) = -\sum_j G_j(t)(V(t) - V_S) + I_a(t) \quad , \qquad \text{(A5)}$$

where G_j is the conductance and V_j is the reversal potential of synapse type *j.* In the main text the case of only G_E and G_I is considered. Here we start from the more general case of arbitrary number of synaptic types.

As noted in (Pokrovskii, 1978), because only V is present in this expression as a state variable of the neuron, we can group the terms into those proportional to V, and free terms, which in turn may be regarded as the two control parameters of the neuron. For convenience, as described in the main text, we introduce these as the total synaptic conductance G and the total synaptic input current I, measured at some arbitrary voltage - as described previously, typically this is taken as the resting potential V_0. The external current may be then rewritten as follows:

$$I_{ext}(t,V) = I(t) - G(t)\,(V(t) - V_0), \qquad \text{(A6)}$$

where the control parameters are:

$$G(t) = \sum_j g_j(t)$$

$$I(t) = \sum_j G_j(t)(V_j - V_0) + I_a \quad . \qquad \text{(A7)}$$

Let's now consider the case of only two types of synapses, excitatory and inhibitory, with the conductances G_E and G_I, and reversal potentials V_E, and V_I. Furthermore, for simplicity we choose $V_I = V_0$, assuming V_0 that is given by the resting potential, which corresponds well with the reversal potential of GABA$_A$ synapses. In this case the control parameters are:

$$G = G_E + G_I \,,\; I = G_E(V_E - V_I)\,. \qquad \text{(A8)}$$

We use these parameters to construct the method for estimation of the conductances G_E , G_I . The reverse transformation of (I, G) into (G_E , G_I) is:

$$G_E = I/(V_E - V_I)$$

$$G_I = G - I/(V_E - V_I) \qquad \text{(A9)}$$

Note that this transformation is simpler than the form given in the main text because of the explicit condition that $V_I = V_0$.

Noise

We also tested the firing-clamp method of conductance estimation for robustness in the case of complex, noise-shaped input, by simulating the input conductances as an Ornstein–Uhlenbeck process (Larkum et al., 2004):

$$G_{E,I}(t+dt) = G_{E,I}(t) + \frac{G^0_{E,I}(t) - G_{E,I}(t)}{\tau} dt + \sigma_{E,I}\, \xi(t) \sqrt{\frac{2\,dt}{\tau}}, \qquad \text{(A10)}$$

where the time constant $\tau = 4$ ms; the dispersion $\sigma_{E,I}$ relative to the amplitude of the time-dependent mean values $G^0_{E,I}$ is equal to 0.2; and the discretization time step dt is 0.05 ms. $\xi(t)$ is the random number with gaussian distribution, zero mean and unit dispersion. Below, we refer $G_E(t) - G^0_E(t)$ and $G_I(t) - G^0_I(t)$ as to the gaussian colored noise.

Parameters

The stimulus parameters used in the firing-clamp method were then analyzed by using the adaptive neuron model. To obtain spike generation at a constant frequency for a wide range of the control parameters I and G, the injected current $I_a(t,V)$ included the meander-like pattern shown in **Figure 1A** of the main text with the following parameters: the amplitudes of the positive and negative steps were 800 pA. The duration of the positive step (τ^+) was fixed to 1 ms, with the negative step duration (τ^-) defined by when the membrane voltage crossed the defined reset value of -70 mV. As explained previously, this reset condition fixes the state of the fast membrane channels, including allowing the neuron to fire at a high rate due to fast sodium channel de-inactivation at the reset voltage. The amplitude of the negative pulse must be high enough to finish the repolarization before the fixed start of the next probe spike over the physiologically meaningful range of the inputs I and G. The frequency of the meander pulses, thus setting the imposed spike interval, was chosen to be 200 Hz, for three main reasons. First, a high frequency is desired to obtain good temporal resolution of the conductance estimation that is commensurate with functionally evoked synaptic inputs. Second, the frequency must also be high enough to avoid "natural" (spontaneous or synaptically evoked) spike generation between the meander-evoked spikes. Finally, the frequency should be low enough to provide time for sodium channel de-inactivation and for integration of the "natural" input current during the interspike intervals, i.e. to provide sensitivity of the voltage to the input current. For most cortical neurons, these constraints imply a frequency range of 80 - 300 Hz. We note that a non-adaptive neuron (e.g. the typical firing characteristic of inhibitory interneurons) can fire at higher rates than a regular-firing (adaptive) neuron (the typical firing characteristic of excitatory neurons), thus implying a higher upper bound for the firing-clamp frequency.

Calibration

Using the adaptive neuron model, we ran a series of simulations over a wide range of the control parameters G and I, setting these parameters constant for each simulation. We calculated and plotted the subthreshold voltage V^{subthr} and the peak voltage V^{peak} as functions of (I, G), as shown in **Supplementary figure 1**. The subthreshold voltage V^{subthr} is defined here as the voltage 1.2 ms preceding the peak of the spike (always before the start of the positive pulse) and the peak voltage V^{peak} as the maximum at spike. These definitions allow measurement of the values directly from the voltage curve, and thus do not require precise

synchronization of recorded voltage and current. The time moment 1.2 ms before the spike peak was optimal for the model, in practice providing a reference time just before spike initiation in all regimes. Note that in the experiments the definitions of V^{subthr} and V^{peak} differ from that in simulations. This change for the experiments was made to reduce the impact of artifacts due to non-zero access resistance (i.e. between recording pipette and cytoplasm) and electrode time constant, which appear during abrupt changes of the injected current in the experimental situation.

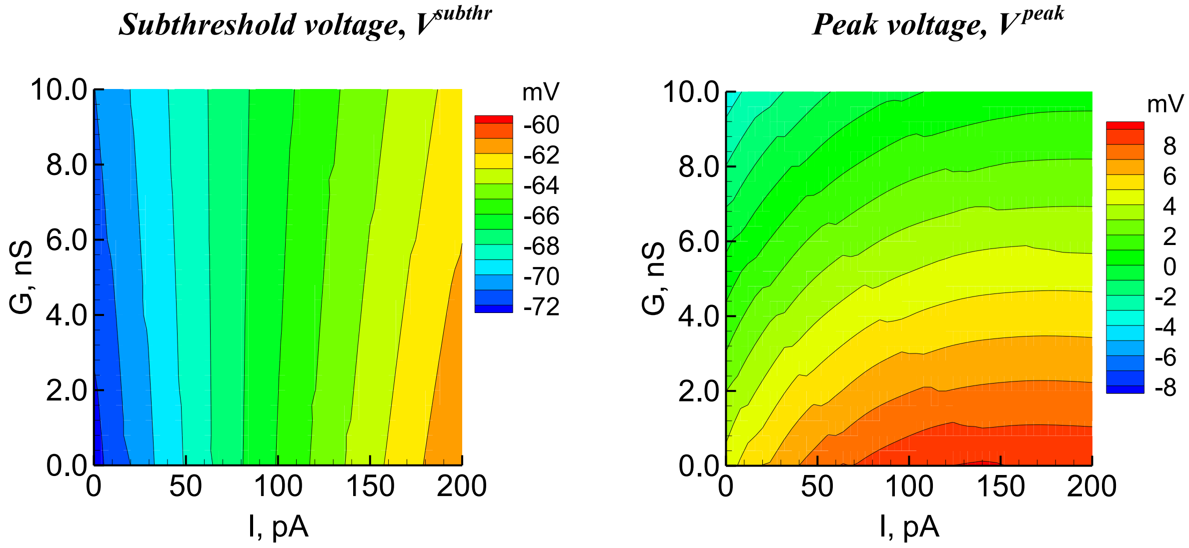

Supplementary figure 1. Dependence of subthreshold voltage and maximum voltage on the control variables I and G in the steady-state regime of spike generation in the model neuron. The meander-like positive-negative current pulses were applied at 200 Hz. The voltage values were measured at the 80th spike.

"Recordings"

Simulated voltage recordings are obtained by generating different traces of $G_E(t)$ and $G_I(t)$ (i. e. the unknown variables in a biological experiment) and applying the same meander-like current $I_a(t,V)$. We measure two parameters (V^{subthr}, V^{peak}) at each probe spike from a given voltage trace, and find the two control parameters (I, G) as a point corresponding to the intersection of the isolines $V^{subthr}=const$, $V^{peak}=const$ of the two plots. The conductances (G_E, G_I) at every spike are then calculated according to the eqs. (A9).

For the input conductances (G_E, G_I) changing in time as shown in **Supplementary figures 2C,D** by slightly varying (red) and highly varying (green) lines, correspondingly, the voltage curve of the model neuron is shown in **Supplementary figures 2A,B**. Measuring the threshold and peak voltages, we estimate the conductances for the peak-to-peak intervals, shown in **Supplementary figures 2C,D** by the dots. In the presence of the noise modeled according to eq. (A10), the estimations are robust and characterized by similar precision (see **Supplementary figure 2E,F**).

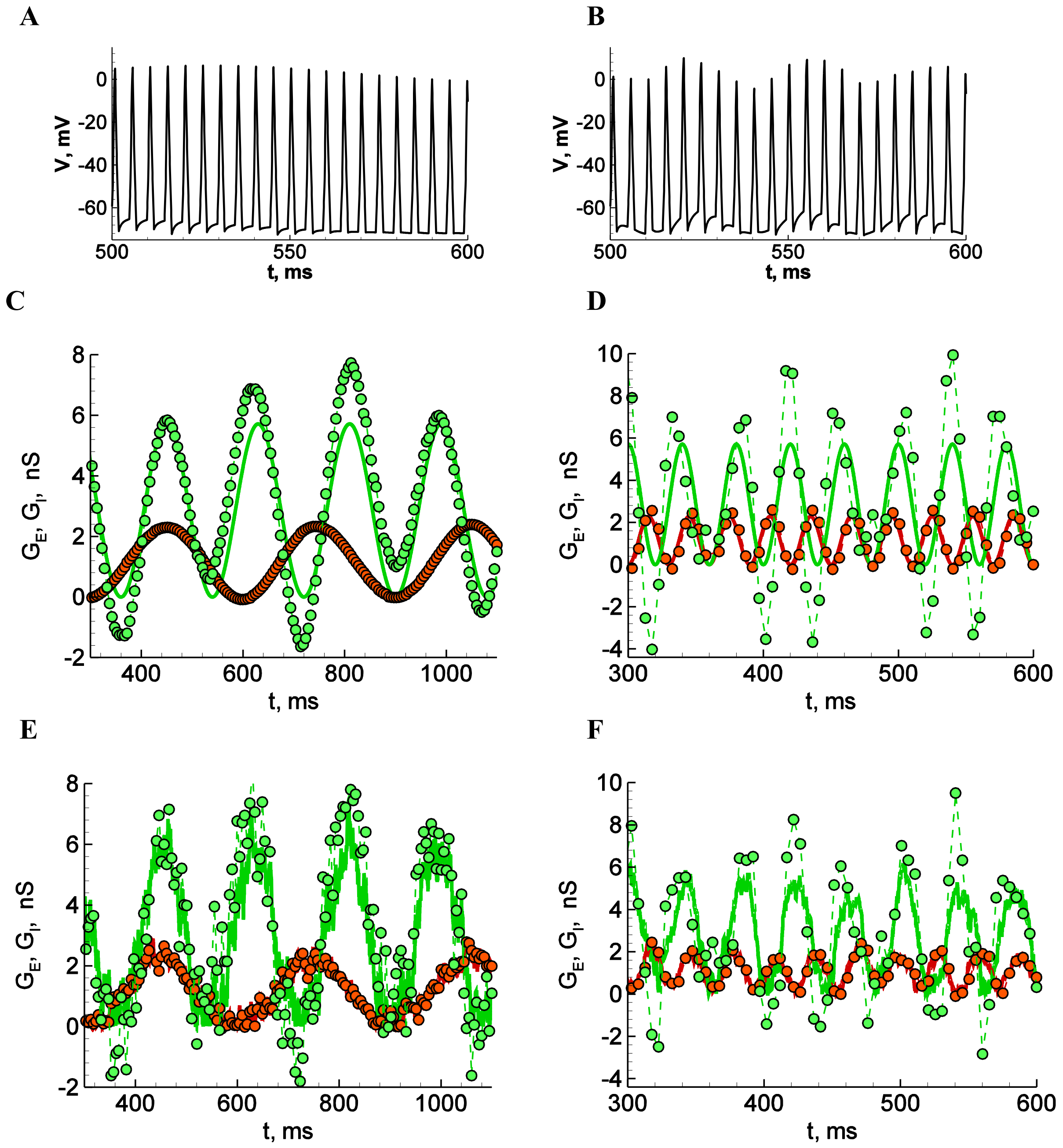

Supplementary figure 2. Estimation of two conductances from "pseudoexperimental" voltage curves. Two examples of excitatory and inhibitory input conductances are shown in **C** and **D** in red and green lines, correspondingly. These input conductances and the injected meander-like current (shown in **Figure 1** of the main text) determine the spike trains shown in **A** and **B**. Estimated points of excitatory and inhibitory conductances are shown by red and green dots, correspondingly. **(E,F).** Estimates in the presence of Gaussian colored noise.

As mentioned, for the experiments we altered the definition of the measured response voltages in order to avoid the contaminating effects of non-zero pipette resistance and capacitance. Specifically, the maximum voltage, V^{peak} was defined as the difference of

potentials measured at the start and at the end of the positive current pulse, thus avoiding electrode artifacts that inevitably follow the abrupt change of injected current. The sub-threshold potential V^{subthr} in the experiments was defined as the mean voltage during inter-meander interval. These variations only slightly affect the estimations.

S3. Estimation of conductances imitated by the dynamic-clamp in a real neuron in a brain slice preparation

To further test the method, we made firing-clamp estimations of artificially injected synaptic conductances supplied by the dynamic-clamp configuration, from a regularly spiking (**supplementary figure 3**) neuron of the rat medial preoptic nucleus recorded in a brain slice preparation.

Methods. The method used has been previously described (Malinina et al., 2010). In short, amphotericin B-perforated patch whole-cell recordings from medial preoptic neurons were made using 150 μm thick acute brain slices from Sprague-Dawley rats and an Axopatch 200B amplifier (Axon instruments, USA). For dynamic-clamp experiments, an acquisition card NI-PCI-6221 (National Instruments, USA) installed in a 2-core Intel processor-based computer running Windows-XP was used. The acquisition card was controlled via custom-built software available at http://www.ioffe.ru/CompPhysLab/AntonV3.htm.

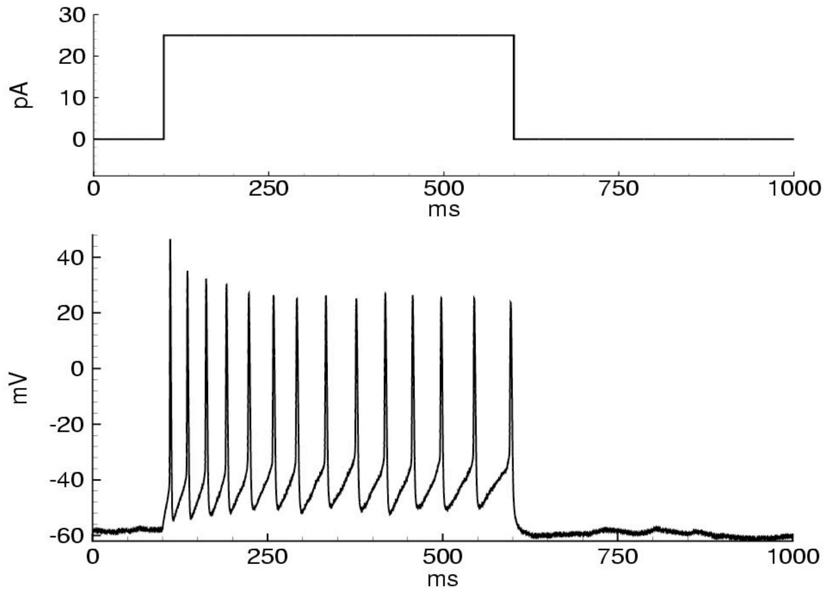

Supplementary figure 3. Impulse activity from a medial preoptic neuron *in vitro*. Voltage response (bottom) to current-step stimulation (top).

"Calibration"

In order to calibrate the firing-clamp method, we measured the functions $V^{subthr}(I, G)$ and $V^{pulse}(I, G)$ by injecting different *I* and *G* (**Supplementary figures 4A,B**). To minimize the number of recordings needed for calibration, we limited the calibrations to the voltage responses to three combinations of a step-wise *G* and a sinusoidal *I*. Two of these conditions are shown in **Supplementary figures 4A,B**. The calibration data for $V^{subthr,appr.}(I, G)$ and $V^{pulse,appr.}(I, G)$ were approximated by cylindrical functions given in **Supplementary figures 4C,D**.

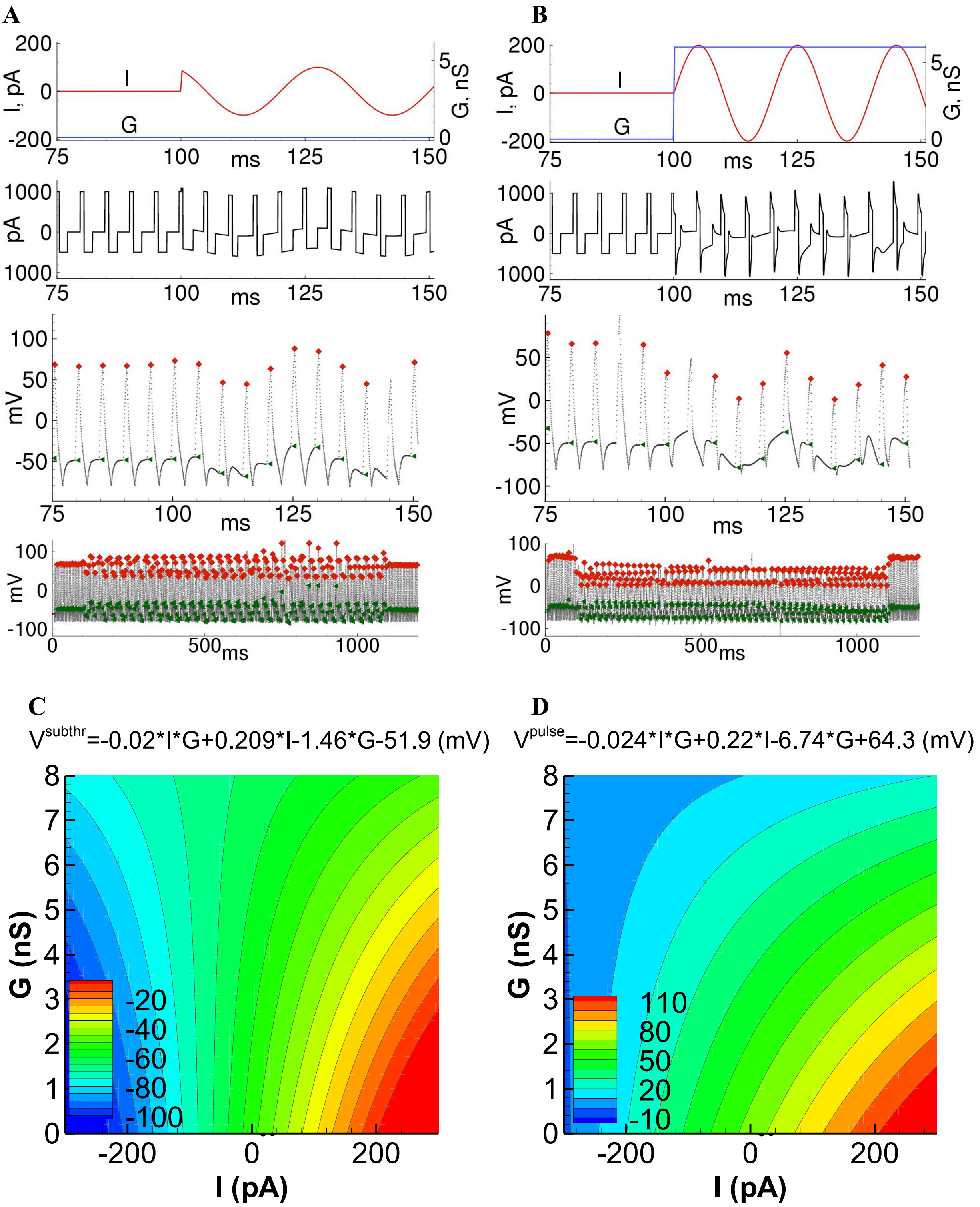

Supplementary figure 4. Calibration records from a medial preoptic neuron *in vitro*. **(A,B)**, Current (2nd from top) and voltage (two lower traces; note different scales for bottom trace) responses to step-wise G (top, blue curve in **B**) and sinusoidal I (top, red curve) (**A**,**B**). Green triangles denote V^{subthr} and red rhomboids V^{pulse}. Bottom curves plotted in different scale present the whole records used for the calibration. (**C**) and (**D**), approximate plots for V^{subthr} and V^{pulse}, reconstructed from the records shown in (**A**,**B**).

“Recordings”

During firing-clamp recordings with the meander-like stimulus current, we applied an artificially generated “synaptic” conductance step G = 6 nS and a sinusoidal current I of amplitude 400 pA and frequency 30 Hz (**Supplementary figures 5A**, lines) and 10 Hz (**Supplementary figures 5B**, lines), using the dynamic-clamp system. These conductance waveforms were then reconstructed by the estimation method described earlier (triangles in **Supplementary figures 5A,B**, top).

Posteriori estimations

At each probe spike i of the recorded voltage shown in **Supplementary figures 5A,B**, $V^{subthr}{}_i$ and $V^{pulse}{}_{\mathrm{i}}$ were measured. Then, the estimates of I_i, G_i were calculated as the solution of the system of equations $V^{subthr,appr.}(I_i, G_i) = V_i^{subthr}$, $V^{pulse,appr.}(I_i, G_i) = V_i^{pulse}$, using the approximations obtained from the calibration. The corresponding values (triangles) are shown in **Supplementary figure 5**. It is seen that the estimations well correspond to the “true” curves.

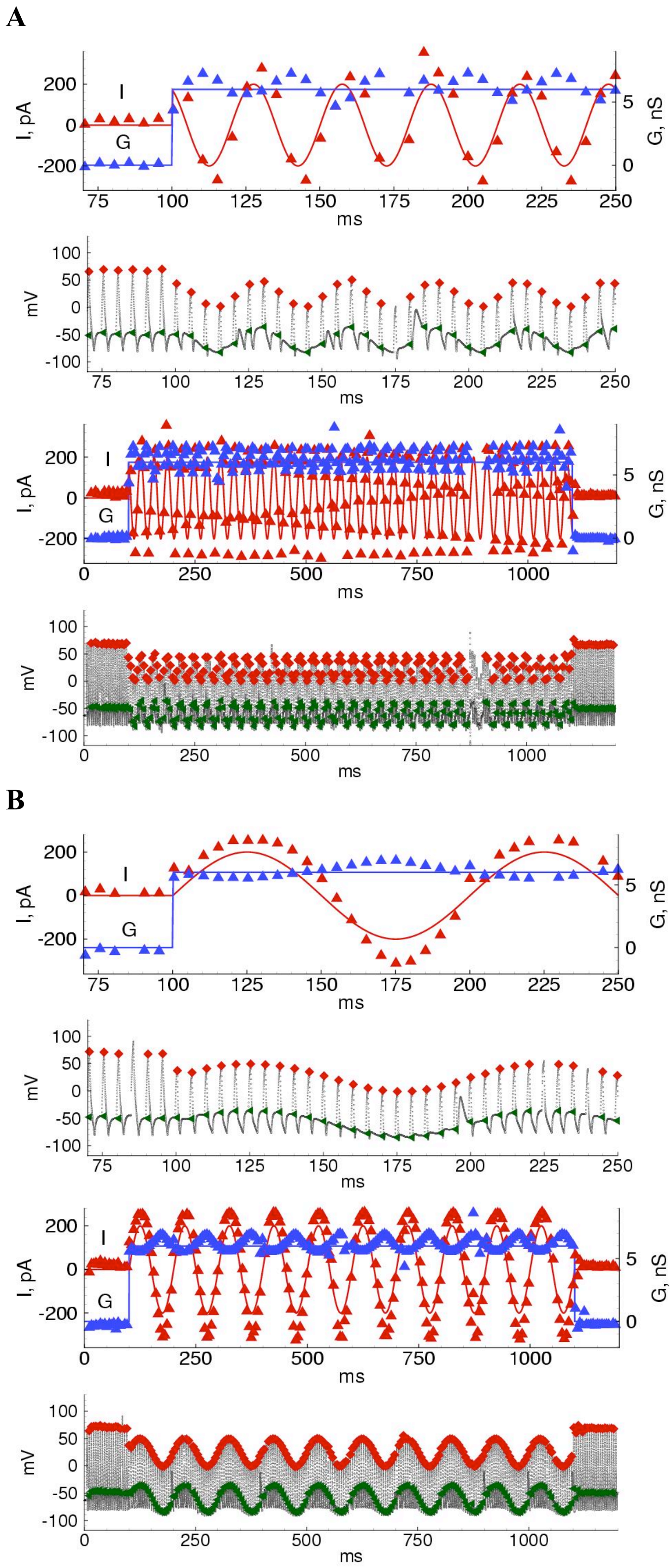
A
B
I, pA
G, nS
I
G
ms
mV

Supplementary figure 5. Recorded data from a medial preoptic neuron in a brain slice preparation. The estimated I and G (red and blue triangles) are compared with the true artificially injected current and conductance (red and blue lines). Recorded voltage curves with the probe spikes are shown as black dotted lines. A step-wise "synaptic" conductance G = 6 nS and a sinusoidal current I with peak-to-peak amplitude 400 pA and frequency 30 Hz (**A**) and 10 Hz (**B**) were applied by the dynamic clamp system. Top two plots in (**A**) and (**B**) show data from the bottom plots at larger magnification.

S4. Evaluation of GABA-ergic reversal potential dynamics

As stated in the main text, whereas the estimation of the total synaptic current I and conductance G does not impose any assumption about the reversal potentials V_E and V_I, the estimation of excitatory and inhibitory synaptic conductances does. The conductances G_E and G_I are obtained from I and G by the formulas (1) of the main text, using certain V_I and V_E. If a synaptic conductance is large, the time course of a synaptic current may significantly alter the corresponding reversal potential, depending on various biophysical properties, including the mechanisms that maintain the concentration gradients of the relevant ions. In particular, this effect may be relevant for the chloride concentration gradient that is the main factor determining the reversal potential of the synaptic current mediated by $GABA_A$ receptors, and, consequently, the estimation of the inhibitory conductance in the present context. In order to account for such factors, we constructed a simple model of the dynamics of the GABA-ergic reversal potential, fitted to the experimental data which allowed a correction for the conductance estimations. Correspondingly, we assume that the synaptic inhibitory current is exclusively composed of chloride ions.

According to Nernst equation and the parameters of our experimental conditions, the reversal potential V_I can be approximated as the chloride reversal potential by the formula:

$$V_I = 25.35 \ln \frac{[Cl]_i}{146}. \tag{1}$$

According to Krishnan and Bazhenov (2011), the chloride concentration dynamics is described by the equation:

$$\frac{d[Cl]_i}{dt} = -\frac{k}{F} I_{Cl} + \frac{[Cl]_i^\infty - [Cl]_i}{\tau_{Cl}}, \tag{2}$$

where $[Cl]_i^\infty$ is the initial chloride concentration, F is the Faraday constant, k is the dimensionless coefficient and τ_{Cl} is the time scale. The synaptic inhibitory chloride current is then given by:

$$I_{Cl} = G_I(V - V_I), \tag{3}$$

where G_I is the GABA-ergic conductance and V is the membrane voltage. In the firing-clamp mode, the main impact of $[Cl]_i$ dynamics to I_{Cl} is during the probe spikes. With respect to the impact on the chloride concentration we assume the shape of the probe spikes to be invariable and that changes of the chloride concentration and inhibitory conductance are much faster than the firing rate. Based on these assumptions, we average the synaptic chloride current over the interspike interval, thus obtaining that I_{Cl} is proportional to G_I with some coefficient k', i.e.

$$I_{Cl}(t) \approx k' G_I(t). \tag{4}$$

Now, introducing (4) into (2), we obtain:

$$\frac{d[Cl]_i}{dt} = -k'' G_I(t) + \frac{[Cl]_i^\infty - [Cl]_i}{\tau_{Cl}}. \tag{5}$$

The formulas (1) and (5) together describe a simple model of V_I dynamics based on two coefficients k'' and τ_{Cl}. The parameter k'' depends on the firing clamp parameters, whereas the time constant τ_{Cl} depends on the properties of ionic transporters and, in the case of whole-

cell and amphotericin-perforated patch recording, on the equilibration of $[Cl]_i$ with the Cl^- concentration used in the recording pipette.

We find the coefficients k'' and τ_{Cl} from fitting (5) to the estimation of V_I in the case where GABA is applied alone, thus $V_I = V_0 + I/G_I$. In the particular case of **Figures 3A** (main text), the coefficients were $k''=0.12\ mM \cdot s^{-1} \cdot nS^{-1}, [Cl]_i^{\infty} = 6.3\ mM$ and $\tau_{Cl}=1\ s$. The time course of G_I and V_I is shown in **Supplementary figure 6**.

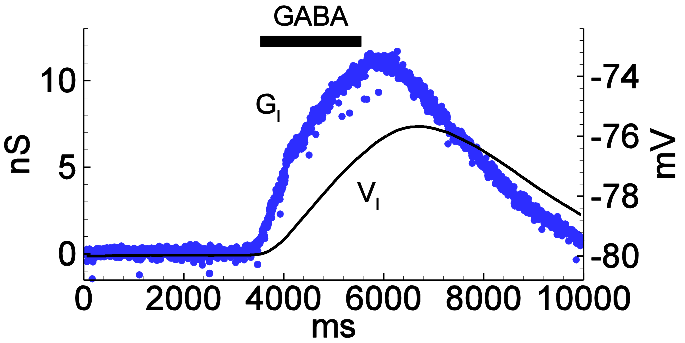

Supplementary figure 6. Estimated inhibitory conductance and inhibitory reversal potential in the case of pure GABA application corresponding to **Figure 3A** of the main text.

S5. Additional example of conductance estimation

Here we present an example of conductance estimations, in addition to those presented in the main text, Figs. 3 and 4. Shown in Suppl. figure 7 is the response to glutamate stimulation for a cell revealing a larger response than that shown in Fig. 3.

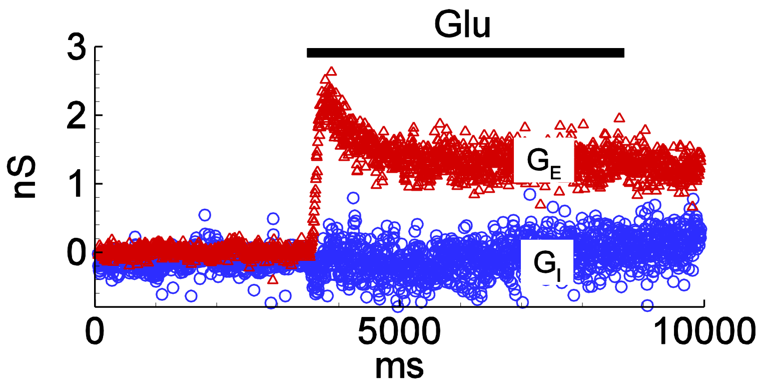

Supplementary figure 7. Estimated evoked conductances in the case of pure glutamate (Glu; 1.0 mM) application. The cell is different from that shown in **Figure 3** of the main text. The resting input conductance of this cell was 0.5 nS.

References

Borg-Graham, L. J., Monier, C., and Fregnac, Y. (1998). Visual input evokes transient and strong shunting inhibition in visual cortical neurons. *Nature* 393, 369–372.

Priebe, N. J., and Ferster, D. (2005). Direction selectivity of excitation and inhibition in simple cells of the cat primary visual cortex. *Neuron* 45(1), 133–145.

Anderson, J. S., Carandini, M., and Ferster, D. (2000). Orientation tuning of input conductance, excitation, and inhibition in cat primary visual cortex. *J. Neurophysiol*. 84, 909–926.

Borg-Graham L. (1998). Interpretations of data and mechanisms for hippocampal pyramidal cell models. In P. S. Ulinski, E. G. Jones, and A. Peters, eds, *Cereb. Cortex*, v.13, pp. 19-138. (Plenum Press, New York).

Kopell, N., Ermentrout, G. B., Whittington, M. A., and Traub, R. D. (2000). Gamma rhythms and beta rhythms have different synchronization properties. *Neurobiology* 97(4), 1867-1872.

Chizhov, A. V., and Graham, L. J. (2007). Population model of hippocampal pyramidal neurons, linking a refractory density approach to conductance-based neurons. *Phys. Rev. E* 75, 011924.

Pokrovskii, A. N. (1978). Effect of synapse conductivity on spike development. *Biofizika*. 23(4), 649-653.

Larkum, M. E., Senn, W., and Lüscher, H.-R. (2004). Top-down dendritic input increases the gain of layer 5 pyramidal neurons. *Cereb. Cortex* 14(10), 1059-1070.

Malinina, E., Druzin, M., and Johansson, S. (2010). Differential control of spontaneous and evoked GABA release by presynaptic L-type Ca^{2+} channels in the rat medial preoptic nucleus. *J. Neurophysiol.* 104(1), 200-209.

Krishnan, G. P., and Bazhenov, M. (2011). Ionic dynamics mediate spontaneous termination of seizures and postictal depression state. *J. Neurosci.* 31(24), 8870-8882.
